# Integrated Enzymatic Membrane Reactor (EMR) for Continuous Production of Antidiabetic, Antihypertensive, and Antioxidant Peptides from Jack Bean

**DOI:** 10.3390/foods15061083

**Published:** 2026-03-19

**Authors:** Rose Uli Ruth Cecilia, Azis Boing Sitanggang, Slamet Budijanto, Endang Prangdimurti

**Affiliations:** 1 Division of Food Science and Technology, Faculty of Engineering and Technology, IPB University, Bogor 16680, Indonesia; 2 Graduate School of Agricultural Science, Tohoku University, Sendai, Miyagi 980-8572, Japan

**Keywords:** ACE inhibitor, antioxidant, bioactive peptides, DPP-IV inhibitor, enzymatic membrane reactor, jack bean

## Abstract

The growing demand for functional foods reflects greater consumer awareness of diet–health links, with bioactive peptides receiving increasing attention for their health-promoting effects. In this study, bioactive peptides exhibiting antioxidant, dipeptidyl peptidase-IV (DPP-IV) inhibitory, and angiotensin-converting enzyme (ACE) inhibitory activities were produced from a jack bean (*Canavalia ensiformis*) protein isolate using a continuous proteolysis system with two enzymes. This study encompassed two major phases: isolating protein from jack beans and implementing a continuous enzymatic hydrolysis process. Key variables examined included the enzyme-to-substrate ratio ([E]/[S]), pH level, and residence time (τ). Optimal performance was achieved at [E]/[S] = 5%, pH = 7.5, and τ = 12 h, yielding a permeate with peptide content of 0.6143 mg SE/mL, along with notable antioxidant capacity and ACE inhibition of 0.0454 mg TEAC/mL and 92.18%, respectively. These results confirm that the jack bean protein isolate is a viable substrate for generating multifunctional bioactive peptides. This study provides a foundation for scalable and sustainable production of functional food ingredients from underutilized legumes using continuous bioprocessing technology. Industrial relevance: Integrating a stirred tank reactor with membrane separation provides a promising approach for continuous bioactive peptide production using a free-enzyme system, helping to streamline processing, reduces the demand for enzyme immobilization, and minimizes batch-to-batch variability. This study shows that continuous hydrolysis of jack bean protein isolate in EMR can enhance antioxidant activity and ACE inhibition of the hydrolysates. This approach offers a safer and more efficient route to support the commercialization of jack bean-based functional products.

## 1. Introduction

In developed countries, increasing awareness of the strong relationship between diet and health has reshaped consumer preferences for functional foods containing bioactive compounds that enhance well-being and reduce disease risks [1,2]. One form of functional foods with potential for future development is bioactive peptides. Consisting of brief protein chains containing 2–20 amino acids, bioactive peptides possess molecular weights under 3 kDa and have attracted growing attention for their diverse physiological functions, including antioxidant, antihypertensive, antidiabetic, antimicrobial, and immunomodulatory activities [3,4,5]. Within intact parent proteins, these peptides exist in a dormant state and only become active once liberated *via* hydrolysis processes.

Food proteins from abundant or underutilized sources are often selected as parent proteins for peptide production, as they can generate valuable bioactivities while supporting the sustainable utilization of agricultural resources [6]. Jack bean (*Canavalia ensiformis*), locally known in Indonesia as Koro Pedang, is an underutilized legume with a high protein content (29.8–32.2%) and is rich in hydrophobic amino acids such as valine, leucine, and phenylalanine—residues often associated with strong antioxidant and ACE inhibitory activities [7,8,9]. Studies conducted previously have demonstrated that peptides derived from jack beans possess multiple beneficial properties, including antioxidant, antimicrobial, DPP-IV inhibitory, and ACE inhibitory capabilities [10,11,12], suggesting their potential as a source of multifunctional bioactive compounds for functional food development.

Peptides can be released from their parent proteins through hydrolysis *via* various mechanisms, including *in vivo* hydrolysis by endogenous gastrointestinal proteases, microbial fermentation, or *in vitro* hydrolysis using specific proteolytic enzymes [13]. The enzymatic approach is preferred due to its environmental sustainability, operational simplicity, and ability to produce high-quality peptides, with several critical factors such as enzyme type, enzyme-to-substrate ratio, pH, temperature, and residence time governing yield and bioactivity [14,15,16].

The enzymatic hydrolysis approach for continuous bioactive peptide production provides notable benefits, including consistent product quality, higher volumetric productivity, shorter cycle times, and lower capital costs [17]. In a previous study, an automated enzymatic membrane reactor (EMR) system was developed [18] to continuously hydrolyze tempe, and its potential for producing functional ingredients, such as bioactive peptides, under controlled flux and residence time, was demonstrated. EMRs have emerged as promising solutions for integrating enzymatic hydrolysis and membrane separation in a single continuous system. This configuration enables the simultaneous reaction and selective removal of peptides while retaining freely suspended enzymes within the reactor, thus enhancing productivity and maintaining steady-state operation [18,19,20]. However, when membrane fouling becomes pronounced, it can reduce flux and overall performance; therefore, operation under constant flux is recommended to sustain a constant production rate [21].

In this study, bioactive peptides were continuously produced from jack bean protein isolate through enzymatic hydrolysis comprising Alcalase and Neutrase within an automated EMR. Both enzymes exhibit specificity toward hydrophobic amino acids, which is favorable for generating peptides with antioxidant, ACE inhibitory, and DPP-IV inhibitory activities [22]. The effects of enzyme-to-substrate ratio, pH, and residence time were evaluated to determine optimal conditions for peptide production and bioactivity, thereby supporting the development of a scalable and sustainable process for functional food ingredient manufacturing.

## 2. Materials and Methods

### 2.1. Materials

Indonesian West Java served as the source for jack beans. Polyethersulfone (PES) flat-sheet membranes featuring molecular weight cut-offs (MWCOs) of 5 kDa (NADIR^®^ UP005) and 4 kDa (NADIR^®^ UH004) were supplied by MANN + HUMMEL (Ludwigsburg, Germany). Novozymes A/S (Bagsværd, Denmark) provided the enzymes Alcalase^®^ 2.5 L (EC 3.4.21.62) and Neutrase^®^ 0.8 L (EC 3.4.24.28). Jayamas Medica Industri (Sidoarjo, Indonesia) supplied the pure water (Water One™, Yamanashi, Japan). NaOH, HCl, K_2_SO_4_, H_2_SO_4_, H_3_BO_3_, methanol pro analysis, sodium dodecyl sulfate (SDS), 2-mercaptoethanol, o-phthalaldehyde (OPA), disodium tetraborate, trichloroacetic acid (TCA), and L-tyrosine were obtained from Merck KGaA (Darmstadt, Germany), which also supplied the Folin–Ciocalteu reagent and Na_2_CO_3_. Sigma-Aldrich Corporation (St. Louis, MO, USA) provided technical-grade bovine milk casein and Trolox. HiMedia Laboratories Private Limited (Thane, India) was the source of 2,2-diphenyl-1-picrylhydrazyl (DPPH). The ACE-Kit WST was obtained from Dojindo (Kumamoto, Japan), while the DPP (IV) Inhibitor Screening Assay Kit was sourced from Abnova (Taipei, Taiwan).

### 2.2. Jack Bean Protein Isolate

Selected jack beans underwent blanching (1:10 *w*/*w*) at 85–95 °C for 10 min, followed by a 72-h soaking period at 25 °C with 12 h intervals between water replacements. After removing and discarding the external bean skin, the beans were subjected to a 15-h drying at 70 °C in a cabinet dryer (Terara Seisakusho C. Ltd., No 4-60SP, Tokyo, Japan). Milling was performed using an FFC-23 pin disc mill (Agrowindo-Maksindo, Blitar, Indonesia), and the material was passed through a 100-mesh screen. Defatting involved treating the flour with technical grade n-hexane (bean-to-hexane ratio = 1:3 *w*/*v*) for 3 h, followed by rinsing and overnight drying in a fume hood for residual hexane elimination. Jack bean protein isolates were obtained through alkaline extraction combined with isoelectric precipitation. Defatted flour was immersed in alkaline water (pH 8.5) (flour-to-water ratio = 1:10 *w*/*v*) for 2 h under continuous agitation. Phase separation was facilitated by centrifugation (Drawell International Technology Ltd., TGL-20MC, Chongqing, China) at 4000× *g* for 10 min. The liquid phase, which contained dissolved proteins, was withdrawn. Its pH was adjusted to reach the protein isoelectric point (pI = ~4.4) using 1.0 N HCl, stirred for 30 min, and centrifuged under identical conditions. The resulting pellet was freeze-dried (Labconco, Kansas City, MO, USA) to yield the protein isolate (T = −47 °C, P = 13 × 10^−3^ mbar, t = 72 h), which was then characterized for moisture, protein, peptide content, antioxidant capacity, and inhibitory activities of DPP-IV and ACE. The crude protein content of the dried material was 95.68% (*wb*), as determined by the official AOAC method and was therefore classified as protein isolate.

### 2.3. Enzyme Filtration

A combination of Alcalase and Neutrase enzymes (1:1 *v*/*v*) underwent filtration through a 5-kDa PES membrane (NADIR^®^ UP005) featuring an effective membrane area of 12.38 × 10^−4^ m^2^ to ensure total enzyme molecule rejection. This enzyme blend was formulated in 0.01 M phosphate buffer (1% *v*/*v*, pH 7.5) and subjected to filtration at a constant flux of 18.17 L/m^2^·h over 4 h. Enzyme activity measurements in the permeate allowed for rejection rate *R* calculation following Maida et al. [23]:.

(1) $$R=\left(\frac{U_{i}-U_{p}}{U_{i}}\right)\times 100\%$$

where $U_{i}$ = the enzyme activity at initial preparation (U/mL); $U_{P}$ = the enzyme activity in the permeate (U/mL).

### 2.4. Continuous Production of Bioactive Peptides Using Automated EMR

**Figure 1:**
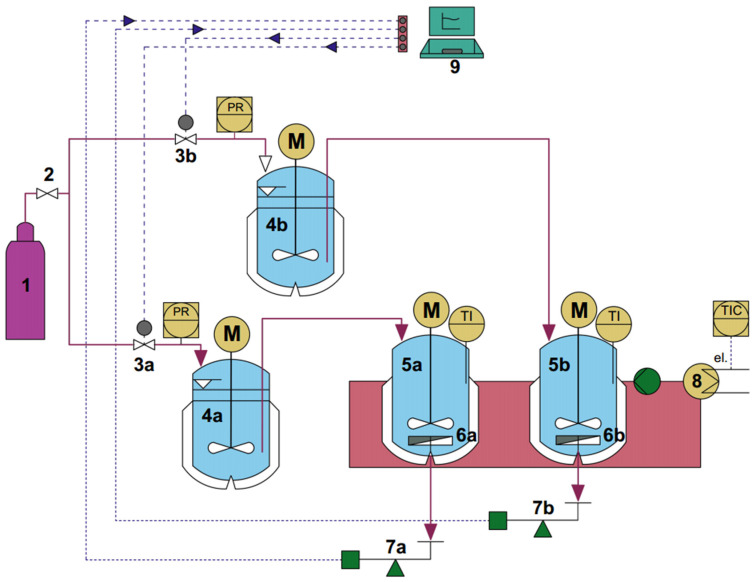
Automated EMR system: (1) nitrogen tank, (2) pressure reducer, (3a-b) PPP-MPPES, (4a-b) substrate tanks, (5a-b) reactors, (6a-b) UF membranes, (7a-b) analytical balances, (8) water bath system, and (9) personal computer (PC) with Lab-VIEW program modified from Sitanggang et al. [18] [M = motor, PR = Pressure recorder, TI = Temperature indicator, TIC = Temperature indicating controller].

An EMR system comprising dual parallel reactors, modified from our earlier research [18] (Figure 1), was employed for continuous hydrolysis to produce bioactive peptides. Data acquisition (DAq) utilized a National Instruments USB-6001 module (NI, Austin, TX, USA) (*9*). Control of a proportional pressure regulator (Festo MPPES-3-1/8-10-010-B, Esslingen, Germany) (*3*) was achieved through programming in the Laboratory Virtual Instrument Engineering Workbench (NI, Austin, TX, USA) (*9*). Pressure supply (*1*) created a gradient between the reactor side (*6*) and permeate side, with the permeate collected on a precision balance (FSRA 320, Fujitsu, Tokyo, Japan) (*7*). A PID controller (*K_c_* = 0.008, *T_I_* = 0.536 min, and *T_D_* = 0.136 min) ensured constant flux throughout the continuous bioactive peptide production. Integration of this controller within the LabVIEW program maintained consistency in the process, as the flux gap between the permeate flux (*J_PV_*) and the target flux (*J_SV_*) remained very small. This allowed the transmembrane pressure (TMP, ∆P) to increase as needed to counteract membrane fouling. Maintaining stable flux enabled this method to support continuous bioactive peptide generation while ensuring consistent residence time.

The jack bean protein isolate was dissolved in 0.01 M phosphate buffer (pH 7.5) at a 0.75 (*w/v*) ratio. The prepared solution was transferred into the substrate tanks (*4a-b*) and subsequently into the reactors (*5a-b*), as shown in Figure 1. The reaction was performed at 50 °C (*8*) under continuous stirring at 300 rpm for 8 h. The effects of operational parameters, including the enzyme-to-substrate ratio ([E]/[S] = 5%, 7.5%, and 12%), pH (7.0, 7.5, and 8.5), and residence time (4, 6, 10, and 12 h), were investigated using single-factor experiments. Permeate samples were collected at specific time intervals (2–8 h) for peptide content, antioxidant activity, DPP-IV, and ACE inhibition analyses. The permeate collected from the containers placed on the analytical balances (*7a-b*) at the end of the reaction was regarded as representative of the overall reaction performance. Samples were collected at intervals, whilst for TMP, and flux were recorded every 5 min. The permeate obtained under the previously optimized operating conditions (i.e., [E]/[S], pH, τ) was further filtered using membranes with smaller MWCOs (4 kDa). A long-term hydrolysis reaction (48 h) was performed under the established optimal conditions. The half-maximal inhibitory concentration (IC_50_) values for the antioxidant and ACE inhibitory activities were subsequently determined. For the SUF (substrate unhydrolyzed–filtered), the jack bean protein isolate was dissolved in buffer solution (pH = 7.5) and then filtered through a 5-kDa membrane under a constant TMP of 2.5 bar. The overall design of hydrolysis reaction to produce bioactive peptides was conducted according to our previous studies [23,24].

### 2.5. Enzyme Activity

The activities of Alcalase and Neutrase were determined, respectively, according to Cupp-Enyard & Aldrich [25] and Rutu et al. [26]. Using a Genesys™ 150 UV-Vis Spectrophotometer from Thermo Fisher Scientific (Waltham, MA, USA), absorbance readings were obtained. The enzymatic activity results were expressed as units/mL (U/mL) and determined through reference to an L-tyrosine standard calibration curve (y = 0.0208x, R^2^ = 0.9968, x = concentration of L-tyrosine (ppm), y = absorbance (-)).

### 2.6. Peptide Content

The peptide content of the permeate was determined using a modified OPA (O-phthalaldehyde) assay based on Wang et al. [27]. OPA reagent preparation involved combining several compounds to achieve a 50-mL final volume: 25 mL of 100 mM disodium tetraborate, 2.5 mL of sodium dodecyl sulphate at 20% (*w*/*w*), 40 mg OPA dissolved in 1 mL methanol, 100 µL β-mercaptoethanol, and 21.4 mL distilled water. Sample aliquots of 150 µL received 3 mL of this OPA reagent, followed by a 4-min dark incubation period at ambient temperature. Absorbance readings were then captured at a-340 nm wavelength with a Genesys™ 150 UV-Vis Spectrophotometer (Thermo Fisher Scientific, Waltham, MA, USA). The peptide content was reported in mg serine equivalent (SE) per mL and quantified using a standard calibration curve of serine (y = 0.2579x, R^2^ = 0.9959, x = concentration of serine (mM), y = absorbance (-)).

### 2.7. Antioxidant Activity

The DPPH (2,2-diphenyl-1-picrylhydrazyl) assay was employed to evaluate antioxidant capacity in both jack bean protein isolate and permeate fractions, following a modified version of the Brand-Williams et al. [28] protocol. DPPH was dissolved in methanol until its concentration reached 78 M to prepare DPPH reagent. A 0.3-mL sample was mixed with 0.7 mL of distilled water. Then, 3 mL of DPPH reagent to the mixture and incubated for 30 min in a dark room at room temperature. The absorbance was measured at a wavelength λ of 515 nm using a Genesys™ 150 UV-Vis Spectrophotometer (Thermo Fisher Scientific, Waltham, MA, USA). Antioxidant activity was reported in mg Trolox equivalent antioxidant capacity (TEAC) per milliliter and quantified using a standard calibration curve of Trolox (y = −0.005x + 0.9042, R^2^ = 0.9924, x = concentration of Trolox (ppm), y = absorbance (-)).

### 2.8. Dipeptidyl Peptidase 4 (DPP-IV) Inhibitory Activity

The measurement of DPP-IV inhibitory activity followed the methodology outlined by Jin et al. [29]. Procedures involved utilizing the DPP-IV Inhibitor Screening Assay Kit in accordance with the protocol specified by the manufacturer. The fluorescence was measured at a wavelength λ of 350–360 nm (excitation) and 450–465 nm (emission) using the SpectraMax^®^ Mini Multi-Mode Microplate Reader (Molecular Devices, San Jose, CA, USA). The inhibitory activity of DPP-IV was calculated as follows (Equation (2)):

(2) $$Inhibitory\;act.\;(\%)=\frac{\left(a-b\right)}{a}\times 100\%$$

where a = fluorescence of the solution containing DPP-IV without the sample (RFU); b = fluorescence of the solution containing DPP-IV and the sample (RFU).

### 2.9. Angiotensin-Converting Enzyme (ACE) Inhibitory Activity

Measurement of ACE inhibitory activity was conducted based on the protocol described by Li et al. [30]. The method utilized the ACE Kit-WST Assay according to the manufacturer’s specifications. A SpectraMax^®^ Mini Multi-Mode Microplate Reader (Molecular Devices, San Jose, USA) measured absorbance at a wavelength of 450 nm. The calculation of ACE inhibitory activity proceeded accordingly (Equation (3)):

(3) $$Inhibitory\;act.\;(\%)=\frac{\left(b-a\right)}{\left(b-c\right)}\times 100\%$$

where a = absorbance of the solution containing ACE and the sample (-), b = absorbance of the solution containing ACE without the sample (positive control) (-), and c = absorbance of the solution without ACE and the sample (reagent blank) (-).

### 2.10. Enzyme Molecular Weight and Charge Distribution

The molecular weight and charge distribution profiles of Alcalase and Neutrase across different pH conditions were computed utilizing http://protcalc.sourceforge.net (The Scripps Research Institute, La Jolla, CA, USA; accessed on 20 April 2025) [18,31].

### 2.11. Statistical Analysis

The results were evaluated statistically using IBM^®^ SPSS^®^ Statistics software, version 25 (IBM, New York, NY, USA). All measurements were performed in four replicates (*n* = 4) and the results are reported as the mean of the four independent measurements. One-way analysis of variance (ANOVA) and post hoc Duncan’s test were employed with a confidence level of 95%.

## 3. Results and Discussion

### 3.1. Jack Bean Protein Isolate Characteristics

Jack beans were processed *via* boiling, soaking, and peeling before being milled into flour—effectively reducing fiber, carbohydrates, and antinutrients like HCN (up to 97.95%), L-DOPA, and trypsin inhibitors [32,33,34] The resulting flour was then defatted to prevent saponification during subsequent NaOH-mediated isolation [35,36].

**Figure 2:**
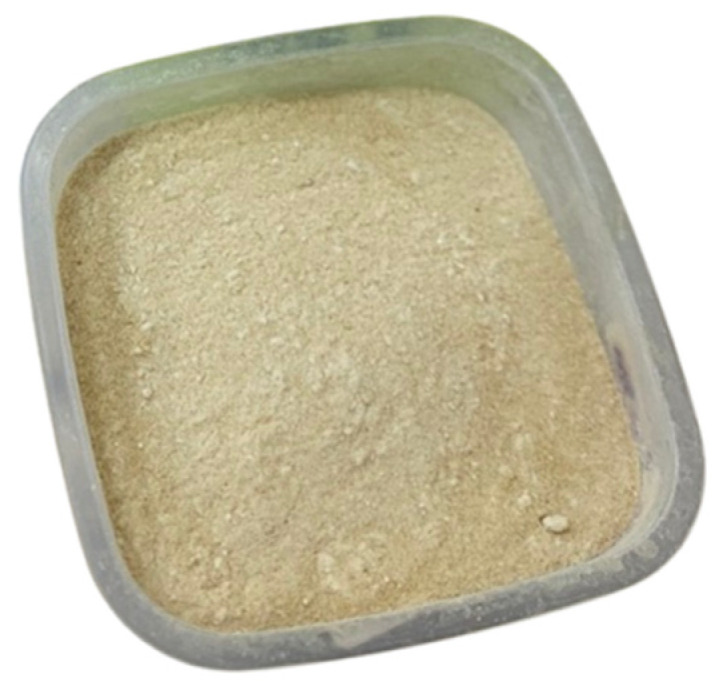
The jack bean protein isolate.

Protein isolation was performed using the AE-IP method for producing high-purity protein isolates [37]. The pH was first adjusted to 8.5 to maximize solubility and then lowered to 4.4 for isoelectric precipitation. The resulting jack bean protein isolate appeared as a fine and nude-colored powder (Figure 2), with a high protein content of 95.68% (*wb*), which surpasses the classification as reported elsewhere [38,39,40], thus qualifying as a protein isolate according to Guéguen et al. [41]. The peptide content, antioxidant capacity, and DPP-IV and ACE inhibitory activities were 55.39 ± 0.29 mg SE/g, 9.01 ± 0.11 mg TEAC/g, 13.44 ± 0.14%, and 78.68 ± 0.13%, respectively.

### 3.2. Enzyme Rejection

The molecular weight of an enzyme is a key consideration in designing EMRs, as the membrane MWCO must be smaller than that of the enzyme to ensure complete retention within the reactor [18]. Insufficient retention can lead to enzyme loss, increased operational costs owing to frequent replenishment, and unwanted hydrolysis of permeate products during storage. In this study, enzyme filtration was performed to assess the rejection efficiency of a 5-kDa PES membrane, which was expected to completely retain the enzymes.

Alcalase (~27 kDa) and Neutrase (~57 kDa) were filtered individually and in combination. The rejection rates obtained were 99.27%, 99.76%, and 99.41%, respectively, slightly below complete retention due to variations in molecular geometry, as MWCOs are based on molecular weight rather than shape [42,43,44]. No synergistic effect was observed between Alcalase and Neutrase; instead, their combined activity decreased, possibly due to non-competitive inhibition (Table 1). As both enzymes achieved >99% rejection, a 1:1 enzyme ratio was selected for subsequent experiments.

Membrane properties, particularly surface charge, must minimize electrostatic interactions to reduce enzyme deposition and fouling, which can increase TMP [31]. During the filtration of Alcalase, Neutrase, and their combination using a 5-kDa PES membrane, a constant flux of 18.17 L/m^2^·h was maintained for 4 h. The TMP increased moderately from about 1.2 to 1.8 bar for the combined enzymes, remaining below the 6-bar reactor limit. Despite minor flux fluctuations, filtration stability and high enzyme rejection (>99%) confirmed that the 5 kDa PES membrane was suitable for the continuous hydrolysis of jack bean proteins and bioactive peptide production.

### 3.3. Effect of Enzyme-to-Substrate Ratio on Production of Bioactive Peptides

The enzyme-to-substrate ratio ([E]/[S]) plays a key role in enzymatic hydrolysis because it directly influences the degree of hydrolysis; an increase in the degree of hydrolysis results from an increase in the enzyme-to-substrate ratio [45,46]. Islam et al. [47] reported that Alcalase and Protamex produced peptides with different degrees of hydrolysis and the results showed a significant correlation with enzyme concentration. The degree of hydrolysis determines the molecular weights of the produced peptides, which in turn influences their bioactivities. A higher degree of hydrolysis leads to more extensive hydrolysis, producing peptides with increased hydrophilicity and solubility [48]. Three enzyme-to-substrate ratios (5%, 7.5%, and 12%) were examined by varying the enzyme concentration and maintaining the substrate concentration.

**Figure 3:**
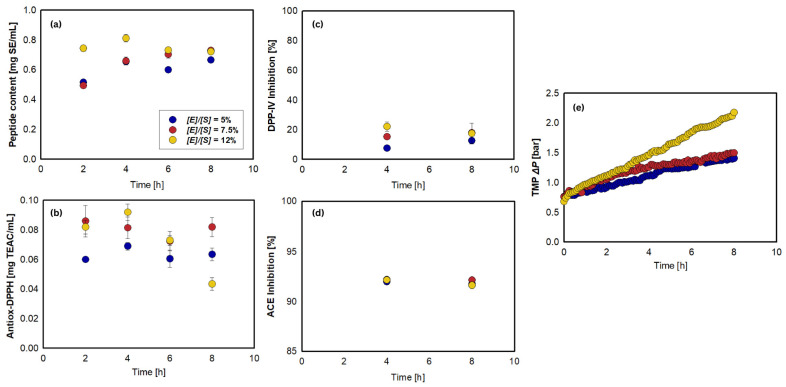
Effect of enzyme-to-substrate ratio on: (**a**) peptide content, (**b**) antioxidant activity, (**c**) DPP-IV inhibitory activity, (**d**) ACE inhibitory activity, and (**e**) TMP during continuous hydrolysis of jack bean protein isolate. Reaction conditions: [S] = 0.75% (*w*/*v*), τ = 6 h (J = 12.12 L/m^2^.h), pH 7.5, N = 300 rpm, T = 50 °C, and 5 kDa PES membrane.

Table 2 (see also Figure 3a–d) presents comparisons of bioactivities between the substrate (unhydrolyzed-filtered) and the cumulative permeates as a function of parameters (i.e., [E]/[S], pH, τ). The highest peptide content was obtained with the 12% treatment, with no significant difference at 7.5%. This indicates that the higher enzyme concentration produced more cleaved peptides and thus more primary amines that reacted with OPA. Antioxidant capacity followed a similar trend, increasing when the treatment of [E]/[S] changed from 5% to 7.5%, then declining at [E]/[S] of 12%, likely due to over-hydrolysis, producing free amino acids with low bioactivities. Peptides are considered more potent antioxidants than free amino acids because of the superior stability of the resulting peptide radicals [49]. Despite a slight rise at [E]/[S] of 7.5%, DPP-IV inhibition showed no significant difference between the substrate and all treatment permeates. In addition, the inhibitory bioactivity of DPP-IV was not very significant in jack beans, and therefore, there was no testing in the next stage of experiments. ACE inhibition was also the highest at 7.5% [E]/[S], but declined at 12% [E]/[S], explaining excessive hydrolysis may degrade bioactive peptides. Herein, optimizing the enzyme-to-substrate ratio is important to prevent the production of over-degraded products.

Figure 3e shows the effect of the enzyme-to-substrate ratio on TMP. TMP tended to increase due to membrane fouling, particularly at the 12% [E]/[S], as foulants accumulated on the membrane surface, reducing flux or requiring higher pressurization to maintain it [50,51]. The PES ultrafiltration carried a negative charge from sulfone groups in its structure [52], and its separation performance was influenced by both steric hindrance and electrostatic interactions [53]. At pH 7.5, Alcalase and Neutrase carried net charges of +0.1 and +2.5, respectively, with isoelectric points (pI) of 7.56 and 8.31 [54]. The negatively charged PES membrane promoted electrostatic attraction with the positively charged enzymes, favoring adhesion [31]. Neutrase, being more positively charged, exhibited a higher tendency to foul the membrane surface, especially as the [E]/[S] increased [24]. However, the TMP values remained similar for the 5% and 7.5% [E]/[S] treatments, suggesting that the charge differences and enzyme concentrations were insufficient to cause major fouling. A significant increase in TMP at 12% confirmed intensified fouling due to higher enzyme loading. Conclusively, the 5% treatment was selected as the optimal treatment owing to its bioactivity performance and TMP values. Even though it had the lowest peptide content, the peptides were able to exhibit high bioactivities.

### 3.4. Effect of pH on Production of Bioactive Peptides

**Figure 4:**
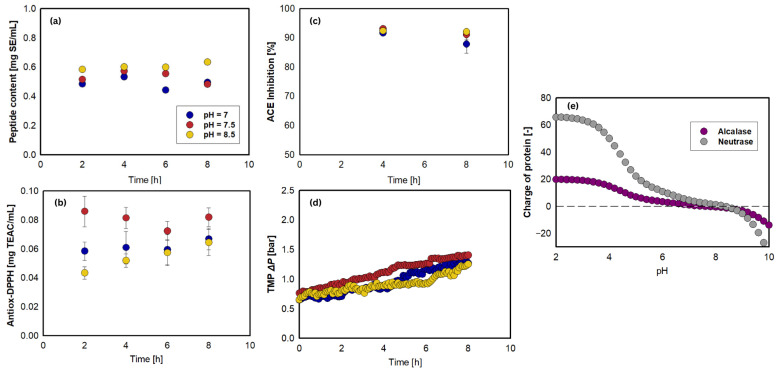
Effect of pH on: (**a**) peptide content, (**b**) antioxidant activity, (**c**) ACE inhibitory activity, and (**d**) TMP during continuous hydrolysis of jack bean protein isolate. Reaction conditions: [S] = 0.75% (*w*/*v*), [E]/[S] = 5%, τ = 6 h (J = 12.12 L/m^2^.h), N = 300 rpm, T = 50 °C, and 5 kDa PES membrane. (**e**) Protein charge distribution of Alcalase and Neutrase at various physiological pH values.

pH significantly influences enzyme activity by affecting protein ionization and stability. Different pH levels during hydrolysis may decrease the proteolytic efficiency of the enzyme, a factor that explains the observed results and contributes to a continuous loss of enzyme activity [55,56]. Table 2 shows (see also Figure 4a–c) that there was no significant difference in antioxidant capacity and ACE inhibition by varying pH, but a significant difference was observed in peptide content. The highest peptide content, antioxidant, and ACE inhibitory activities were observed at pH 7.5. According to the enzyme manufacturer (Novozymes A/S, Bagsværd, Denmark), Alcalase^®^ 2.5 L functions optimally at pH 7–10, whereas Neutrase^®^ 0.8 L operates best at pH 7.0. Previous research indicates that Alcalase performs best in alkaline conditions (>pH 8), whereas Neutrase performs best at pH 5.5–7.5 [57,58]. Therefore, pH 7.5 may serve as a compromise between the two enzymes preferences, creating an environment that enhances their combined activity [22].

Figure 4d shows the effect of pH on the TMP. As mentioned previously, at pH 7.5, Alcalase and Neutrase carried net charges of +0.1 and +2.5, respectively. As the pH increased, protein molecules became more negatively charged, thereby reducing the electrostatic attraction between enzyme molecules and the negatively charged membrane surface. This shift led to greater electrostatic repulsion, resulting in slightly lower TMP at higher pH values (pH 8.5) compared to pH 7.0 and 7.5. However, the difference in TMPs among these treatments was minimal, as the change in enzyme charge within the pH 7.0–8.5 range was relatively small (see Figure 4e). Conclusively, treatment at pH 7.5 emerged as the preferred condition, attributed to its superior bioactivity profile and compatibility with the acidic requirements of both enzymes.

### 3.5. Effect of Residence Time on Production of Bioactive Peptides

**Figure 5:**
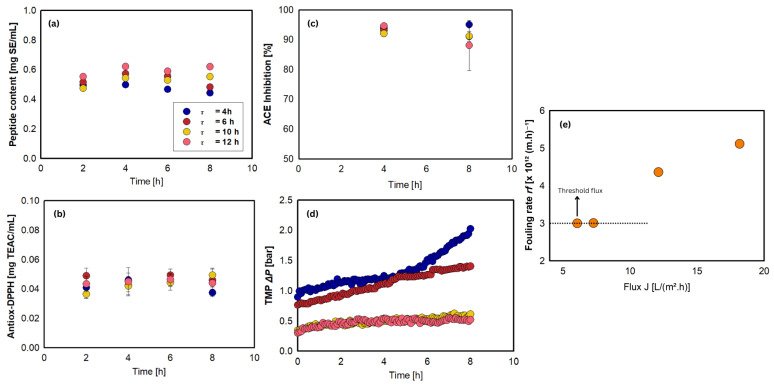
Effect of residence time τ: on (**a**) peptide content, (**b**) antioxidant activity, (**c**) ACE inhibitory activity, and (**d**) TMP during continuous hydrolysis of jack bean protein isolate. Reaction conditions: [S] = 0.75% (*w*/*v*), [E]/[S] = 5%, τ = 6 h (J = 12.12 L/m^2^.h), N = 300 rpm, T = 50 °C, and 5 kDa PES membrane. (**e**) Determination of threshold flux based on fouling rate $r_{f}$ ($r_{f}=\frac{d\left(∆P\right)}{dt}$) for different permeate fluxes.

Residence time refers to the duration for which the substrate stays in the reactor, directly influencing how long the enzymes interact with the substrate. Determining the optimal residence time is essential to prevent both incomplete and excessive hydrolysis [59]. Therefore, a longer residence time generally leads to a greater extent of hydrolysis and an increased peptide yield (Table 2; see also Figure 5a–c). Despite this, residence time did not appear to significantly influence antioxidant capacity (except for τ = 6 h) or ACE inhibitory activity. This suggests proteolysis had already reached its maximum extent at the given enzyme-to-substrate ratio (i.e., [E]/[S] = 5%), causing excessive breakdown of the hydrolysis products. It is widely recognized that bioactive peptides tend to be small in size [60]; thus, a higher degree of hydrolysis is typically favorable. Nevertheless, when hydrolysis proceeds to an excessive extent, it may result in the formation of free amino acids rather than peptides.

Fluctuations were observed in the peptide content, with the highest value found at τ of 12 h. The longer residence time resulted in a significantly higher peptide content than the other shorter residence times. These conditions facilitate enhanced proteolytic breakdown, enabling low-molecular-weight peptides to penetrate the membrane more effectively. Thus, it can be concluded that the amount of peptide produced is high, with insignificant antioxidant activity and ACE inhibition. The variability in protein cleavage patterns by enzymatic action likely explains this observation. Endopeptidase generates peptides with heterogeneous chain lengths [61]. These peptides produced over time may differ in size, structure, or sequence, resulting in varying bioactivity.

Figure 5d shows the impact of residence time on TMP. Ahorter residence time (corresponding to a higher flux) caused a sharp increase in TMP, likely due to enhanced convective transport that promotes fouling on the membrane surface, a pattern observed in previous studies [62,63]. Nevertheless, the constructed EMR demonstrates pressure tolerance reaching 6.0 bar, suggesting that extended reaction durations are practical for bioactive peptide generation. To address this, the threshold flux concept was applied to define the boundary between low- and high-fouling conditions. According to Figure 5d, at a residence time of 12 h, only a slight increase in TMP was observed, indicating minimal fouling. This flux can therefore be considered the threshold flux, representing the point at which the fouling rate begins to rise with increasing flux [19].

### 3.6. Long-Term Continuous Bioactive Peptide Production

**Figure 6:**
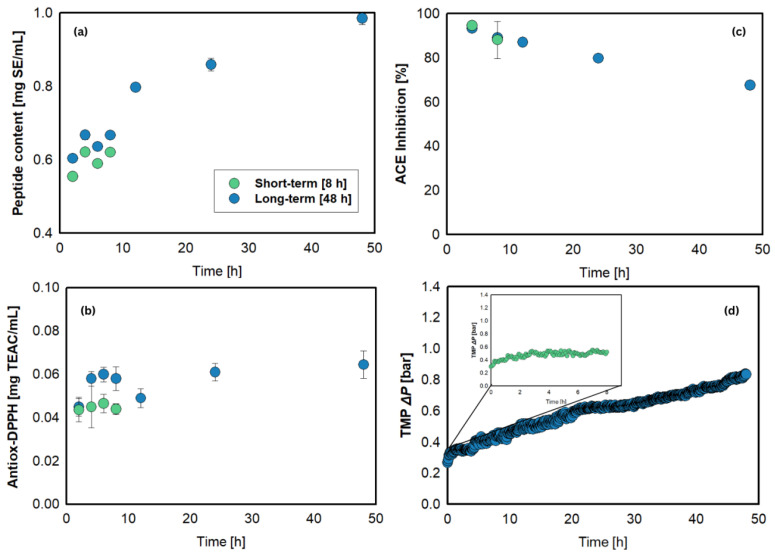
Long-term operation under optimum operating conditions. Profile of (**a**) peptide content, (**b**) antioxidant activity (DPPH assay), (**c**) ACE inhibitory activity, and (**d**) transmembrane pressure (TMP). Reaction conditions: [S] = 0.75% (*w*/*v*), *[E]/[S]* = 5%, τ = 12 h (Flux J = 6.06 L/m^2^.h), pH = 7.5, N = 300 rpm, T = 50 °C, and 5- kDa PES membrane.

A long-term experiment was conducted under previously optimized conditions ([E]/[S] = 5%, pH 7.5, and residence time *τ* = 12 h) (Figure 6a–d). During the 48 h continuous reaction, the peptide content increased; meanwhile, minor variations were noted in the antioxidant capacity measurements. The slight fluctuations in antioxidant activity may be attributed to variations in peptide sequences generated during hydrolysis, as certain peptide sequences tend to possess antioxidant properties. Despite that, both results indicated that the enzyme was still working to hydrolyze the protein into peptides. However, a similar trend was not observed in ACE inhibitory activity, which potentially indicates that the resulting peptides tend to exhibit antioxidant properties. It is important to note that bioactivities do not solely depend on the molecular weight of peptides but also on their sequence. Enhanced ACE inhibitory activity is typically associated with peptides containing aromatic or cyclic amino acids (P, Y, W, F) and positively charged residues (R, K) positioned at the C-terminus, as well as those featuring aliphatic chains (e.g., G, I, L, V) at the N-terminus, since these amino acids strengthen the binding affinity between peptides and ACE [64,65,66]. Furthermore, research has demonstrated that hydrophobic (L, V, A, P, F), aromatic (Y, W, H), sulfur-containing (C, M), acidic (E), and basic amino acids (K) play beneficial roles in promoting antioxidative properties [67,68,69]. Therefore, further investigations are needed to characterize the structure and sequence of peptides produced from hydrolysis, which will support the establishment of long-term reaction optimization strategies.

Over the course of the operation, the TMP gradually increased from approximately 0.3 bar to 0.8 bar. Based on this rise of approximately 0.01 bar/h, it was estimated that the continuous process could be sustained for up to 480 h (~20 days) before reaching the system’s maximum pressure tolerance of 6.0 bar. However, the projected operational time must also consider the potential decline in the enzyme activity within the reactor. If enzyme deactivation occurs, supplementary enzyme addition is necessary to maintain consistent performance, as previously reported [31]. The constant flux operation and relatively stable antioxidant activity observed during the long-term operation indicate that the developed EMR system is feasible for continuous protein hydrolysis, with further investigations, as mentioned above, required to optimize the ACE inhibition properties.

### 3.7. IC_50_ Values for Antioxidant and ACE Inhibition of Peptide Fractions

**Figure 7:**
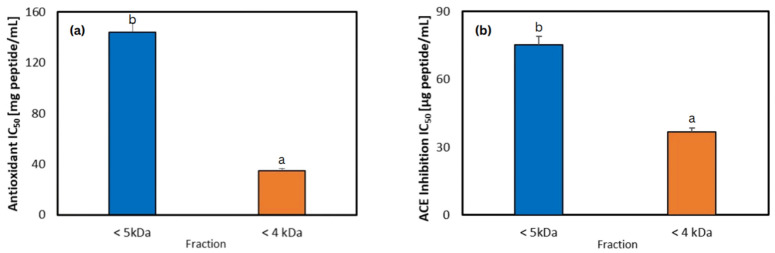
IC_50_ values of (**a**) antioxidant and (**b**) ACE inhibitory activities. Different superscript letters indicate significant differences with confidence level of 95%.

An increase in bioactivity (indicated by lower IC_50_ values) was observed with the use of membranes with smaller molecular weight cut-offs, consistent with trends reported in our previous work [70]. The <4 kDa peptide fraction showed higher levels of bioactivity, with IC_50_ values of 34.93 mg peptide/mL for antioxidant capacity and 36.62 µg peptide/mL for ACE inhibition, compared with the <5 kDa fraction (Figure 7a,b). These results align with previous studies reporting that the lowest-molecular-weight fraction was frequently the fraction with the best antioxidant activity and ACE inhibition [18,60,70,71].

In this study, the IC_50_ value for antioxidant capacity was generally higher than those reported in previous studies on jack bean and other legume-derived protein hydrolysates [18,24,72,73,74,75,76,77]. Meanwhile, the IC_50_ value for ACE inhibition was lower compared to most other studies [18,78,79,80,81], except those that used <2 kDa membrane filtration [24,72]. Hence, the permeates obtained under the optimal reaction conditions followed by the long-term reaction indicate stronger ACE inhibitory activity compared to antioxidant activity. These findings support the idea that the most effective ACE inhibitory peptides are typically of low molecular weight. Nonetheless, reducing the peptide size does not necessarily lead to greater bioactivity or lower IC_50_ values. Sitanggang et al. [18] noted that when peptides are below 1 kDa, the relationship between molecular weight and ACE inhibitory activity becomes weak. Instead, peptide structure, particularly amino acid composition and sequence, plays a more decisive role.

## 4. Conclusions

A continuous bioactive peptide production from jack bean protein isolate was achieved using an automated EMR. The optimized parameters consisted of a 5% enzyme-to-substrate ratio, pH 7.5, residence time of 12 h, and a 5-kDa PES membrane. The resulting permeate had a peptide content of 0.61 mg SE/mL, antioxidant activity of 0.04 mg TEAC/mL, and ACE inhibition of 92.18%. Further fractionation of the long-term permeate using a 4-kDa membrane enhanced both antioxidant and ACE inhibitory activities, with the <4 kDa fraction showing the highest bioactivities—34.93 mg peptide/mL for antioxidant capacity and 36.62 µg peptide/mL for ACE inhibition. In addition, long-term continuous operation confirmed that the EMR system could maintain stable performance, as indicated by the consistent flux and relatively steady antioxidant activity. However, further investigations are needed regarding peptide sequences to optimize ACE inhibitory activity, verification of the *in vivo* activity of concanavalin peptides, and improvements in DPP-IV inhibitory activity to enhance their potential as multifunctional functional food components.

## Figures and Tables

**Table 1:** Enzyme activity profiles of Alcalase, Neutrase, and their combination.

	Activity (U/mL)	Rejection Rate (%)
Alcalase (1%)	31,835.98 ± 25.20 ^c^	99.27
Alcalase (5-kDa permeate)	232.80 ± 54.93 ^a^	
Neutrase (1%)	49,179.89 ± 792.15 ^d^	99.76
Neutrase (5-kDa permeate)	116.40 ± 70.16 ^a^	
Alcalase-Neutrase (1%, 1:1 *v*/*v*)	8593.75 ± 69.96 ^b^	99.41
Alcalase-Neutrase (5-kDa permeate)	116.35 ± 5.29 ^a^	

Note: Different superscript letters in a column show significant differences with a confidence level of 95% (*p* < 0.05).

**Table 2:** Characteristics of substrate and cumulative permeate. Reaction conditions for different treatments are given in Figure 2, Figure 3 and Figure 4.

Treatment		Peptide Content (mg SE/mL)	Antioxidant Capacity (mg TEAC/mL)	ACE Inhibition (%)	DPP-IV Inhibition (%)
Substrate UF		0.0257 ± 0.0020 ^ab^	0.0484 ± 0.0037 ^bc^	63.24 ± 1.51 ^c^	7.12 ± 0.12 ^a^
Permeate, [E]/[S]	5%	0.4703 ± 0.0102 ^cd^	0.0539 ± 0.0034 ^cde^	90.39 ± 0.66 ^d^	14.62 ± 7.72 ^a^
	7.5%	0.7105 ± 0.0256 ^h^	0.0624 ± 0.0028 ^e^	92.11 ± 0.39 ^d^	17.96 ± 3.15 ^a^
	12%	0.7243 ± 0.0198 ^h^	0.0589 ± 0.0044 ^de^	91.91 ± 0.27 ^d^	10.16 ± 3.92 ^a^
Substrate UF	7.0	0.0318 ± 0.0026 ^b^	0.0424 ± 0.0043 ^ab^	9.59 ± 0.18 ^a^	-
	7.5	0.0211 ± 0.0021 ^ab^	0.0484 ± 0.0037 ^bc^	12.31 ± 0.60 ^a^	-
	8.5	0.0113 ± 0.0021 ^a^	0.0354 ± 0.0038 ^a^	27.43 ± 0.80 ^b^	-
Permeate, pH	7.0	0.4789 ± 0.0083 ^d^	0.0549 ± 0.0070 ^cde^	89.51 ± 0.99 ^d^	-
	7.5	0.5257 ± 0.0077 ^f^	0.0634 ± 0.0042 ^e^	90.39 ± 8.61 ^d^	-
	8.5	0.5128 ± 0.0139 ^ef^	0.0584 ± 0.0103 ^de^	90.68 ± 6.55 ^d^	-
Substrate UF		0.0211 ± 0.0021 ^ab^	0.0469 ± 0.0077 ^bc^	12.30 ± 0.60 ^a^	-
Permeate, residence time τ	4 h	0.4554 ± 0.0023 ^ac^	0.0459 ± 0.0044 ^bc^	96.06 ± 0.67 ^ad^	-
	6 h	0.5257 ± 0.0077 ^f^	0.0634 ± 0.0042 ^e^	90.39 ± 8.61 ^d^	-
	10 h	0.5021 ± 0.0117 ^e^	0.0509 ± 0.0112 ^bcd^	90.55 ± 0.00 ^d^	-
	12 h	0.6143 ± 0.0064 ^g^	0.0454 ± 0.0060 ^bc^	92.18 ± 1.83 ^d^	-

Note: Different superscript letters in a column show significant differences with a confidence level of 95% (*p* < 0.05).

## Data Availability

The original contributions presented in this study are included in the article. Further inquiries can be directed to the corresponding author.
